# Characterisation and risk factors of astigmatism among children and adolescents aged 3–20 years in Northwestern China

**DOI:** 10.7189/jogh.16.04047

**Published:** 2026-02-06

**Authors:** Jun-han Wei, Yi-ming Guo, Jia-qi Wang, Yi-jin Han, Qian Yao, Guo-yun Zhang, Lu Ye

**Affiliations:** Shaanxi Eye Hospital, Xi’an People’s Hospital (Xi’an Fourth Hospital), Affiliated People’s Hospital of Northwest University, Xi’an, Shaanxi, China

## Abstract

**Background:**

Astigmatism is a common refractive error in children and adolescents. This study aimed to investigate the prevalence, severity, types, correction status, and associated risk factors of astigmatism among children and adolescents in Shaanxi Province, China.

**Methods:**

A cross-sectional study was conducted involving 236 397 children and adolescents aged 3–20 years from Shaanxi Province, selected through multistage stratified cluster random sampling. Demographic information was gathered via structured questionnaires, and refractive status was assessed using an autorefractor without cycloplegia. Astigmatism was defined as cylindrical refractive error ≥0.50 dioptres.

**Results:**

The overall prevalence of astigmatism was 73.81% among the studied population. Age showed a clear positive association with prevalence, which was significantly higher in adolescents compared to younger children. Similar patterns were observed across advancing educational levels. Males showed slightly higher prevalence than females. Geographic distribution revealed a north-to-south gradient, with northern regions having the highest prevalence. Regarding astigmatism types, with-the-rule astigmatism and compound myopic astigmatism were the predominant forms. Only 30.73% of affected subjects had received refractive correction, with correction rates markedly increasing with age and educational level. Multivariate analyses confirmed that older age, higher educational attainment, male gender, northern residence, and non-Han ethnicity were independent risk factors for astigmatism.

**Conclusions:**

Astigmatism prevalence among children in Shaanxi Province is considerably higher than previously reported in other Chinese regions, with significant variations across demographic factors. The low correction rate, particularly among younger children, highlights the need for enhanced early screening and timely intervention programmes to prevent visual function impairment.

Astigmatism is a common refractive error arising from unequal refractive power across different ocular meridians, resulting in asymmetric refraction, frequently occurring alongside myopia and hyperopia [1]. This optical condition results when the cornea, lens, or both exhibit asymmetric curvatures, causing light rays to focus at multiple points rather than converging at a single focal point on the retina [2]. Uncorrected astigmatism impairs visual acuity [3–5], and when present during critical developmental periods, is associated with amblyopia [6,7], contrast sensitivity loss [8], and impaired stereopsis [9]. Evidence also indicates that the presence of astigmatism is associated with myopia prevalence and progression [10–13].

Among Chinese children and adolescents, the prevalence of astigmatism exhibits considerable geographic heterogeneity, with reported rates varying from 12.9–61.7% across different regions [9,12–24]. These regional disparities likely reflect differences in ethnic composition, living environments, and lifestyle habits. The precise ethology of astigmatism remains incompletely elucidated, though current evidence suggests a complex interaction between hereditary predisposition and environmental exposures. Prior research identifies multiple contributing factors, including demographic variables (age, ethnicity, race), developmental conditions, genetic variants, anatomical features (eyelid configuration and tension, extraocular muscle forces), and lifestyle elements (tobacco exposure, illumination conditions, screen time) [2,4,13,18,25–30]. Certain ocular conditions, including keratoconus, pellucid marginal degeneration, pterygium, and corneal surgery, can also induce or exacerbate astigmatic refractive errors through corneal surface alterations [31–34].

Although several studies have described the epidemiology of astigmatism in children and adolescents across different regions of China, and Zhang et al. reported an astigmatism prevalence of 59.3% in Xi'an, the provincial capital of Shaanxi Province [16], comprehensive epidemiological data encompassing diverse geographic areas and populations across Shaanxi Province remain markedly limited. Understanding regional variation within Shaanxi Province is essential, as the province's substantial north-south span encompasses diverse geomorphological types, with constituent regions demonstrating significant disparities in socioeconomic development, lifestyle, and environmental factors. Large-scale epidemiological investigations can comprehensively characterise astigmatism distribution and provide regional evidence for understanding environmental and socioeconomic influences on refractive development in children and adolescents. Furthermore, given that refractive errors are a leading cause of paediatric visual impairment globally, epidemiological data from regions with varied geographic and economic backgrounds hold substantial value for refining global prevention and control strategies.

Accordingly, this study examines children and adolescents aged 3–20 years to estimate the prevalence, magnitude, and distribution of astigmatism types, assess the status of refractive correction, and explore potential risk factors in this regional population. The findings aim to provide an evidence base for the prevention and management of paediatric refractive errors and to inform strategies that promote comprehensive eye health in children.

## METHODS

### Study participants

From May to July 2021, we conducted a cross-sectional study involving 266 306 children from 641 schools across Shaanxi Province, China, using a multistage stratified cluster random sampling method. The study covered all cities in the province except Tongchuan. Based on geographical location, we stratified the cities into three regions: Central Shaanxi (Xi'an, Baoji, Xianyang, and Weinan), Northern Shaanxi (Yan'an and Yulin), and Southern Shaanxi (Hanzhong, Ankang, and Shangluo).

The inclusion and exclusion criteria were as follows:

(1) age between 3–20 years

(2) absence of significant ocular diseases such as strabismus, cataracts, glaucoma, or eye trauma

(3) no history of ocular surgery

(4) no use of contact lenses within the previous three months

(5) no systemic or mental disorders that could affect outcome assessment.

After screening and excluding participants with incomplete information, a total of 236 397 children and adolescents were included in the final analysis. This study adhered to the principles of the Declaration of Helsinki and received approval from the Ethics Committee of Xi'an People's Hospital (Xi'an Fourth Hospital) (approval number 20210053). Written informed consent was obtained from local education departments, schools, teachers, and parents following a comprehensive explanation of the study procedures. Additionally, legal guardians of all participating children provided signed informed consent forms. The study is reported in accordance with the Strengthening the Reporting of Observational Studies in Epidemiology (STROBE) guidelines (STROBE checklist in the **Online Supplementary Document**).

### Study examinations and questionnaire

All examinations were conducted in standardised settings within the school premises by optometrists or ophthalmic technicians who had received comprehensive standardised training. All children underwent visual acuity and refractive examinations. During the examination period, a structured questionnaire was administered face-to-face to collect basic information including age, sex, education level, ethnicity, medical history, history of refractive correction, and correction methods. Uncorrected visual acuity was measured using a standard logarithmic visual acuity chart at a distance of five meters. Measurements were taken first for the right eye, followed by the left eye, and recorded in LogMAR format. Refractive measurements were performed using an autorefractor (KR-800; Topcon Co., Tokyo, Japan) without cycloplegia. Three measurements were taken for each eye, starting with the right eye followed by the left. If the difference between any two readings exceeded 0.50 dioptres (D), additional measurements were taken. The mean value of the final consistent measurements was recorded.

To avoid inter-eye correlation and to ensure consistency, only the results from the eye with the higher absolute cylindrical value were included in the analysis, following the approach used in previous epidemiological studies on refractive error [35–37]. Astigmatism was defined as a difference of ≥ 0.5 D between the two principal meridians of the eye, *i.e.* cylinder (CYL) ≥ 0.5 D. Astigmatism was classified according to different criteria as follows: by degree of astigmatism: mild astigmatism (0.50 ≤ CYL < 1.00 D), moderate astigmatism (1.00 ≤ CYL < 2.00 D), and high astigmatism (CYL ≥ 2.00 D); by the relationship of the two principal meridians to the retina: simple myopic astigmatism (one focal line in front of the retina, one on the retina), simple hyperopic astigmatism (one focal line behind the retina, one on the retina), compound myopic astigmatism (both focal lines in front of the retina with different refractive powers), compound hyperopic astigmatism (both focal lines behind the retina with different refractive powers), and mixed astigmatism (one focal line in front of the retina, one behind); by the axis of the maximum refractive power: against-the-rule astigmatism (maximum refractive power at 90° ± 30°), with-the-rule astigmatism (maximum refractive power at 150–180° or 0–30°), and oblique astigmatism (maximum refractive power at 30–60° or 120–150°) [38]. Spherical equivalent (SE) was calculated as the spherical refractive error plus half the cylindrical refractive error (SE = Sphere + Cylinder/2).

### Data analysis

Statistical analyses were performed using SPSS 23.0 (IBM Inc., Armonk, NY, USA) and MATLAB R2016a (Mathworks, Natick, MA, USA). The Kolmogorov-Smirnov test was used to assess the normality of continuous variables. Non-normally distributed continuous data were presented as median (interquartile range (IQR)), and categorical variables were expressed as number (percentage). Kruskal-Wallis H tests were conducted to analyse differences in visual acuity and refractive parameters across age groups, education levels, and geographical regions, while Mann-Whitney U tests were employed to examine differences between genders and ethnicities. χ^2^ tests were performed to analyse the differences in astigmatism severity, classification distribution, and refractive correction status across different groups (age, education level, gender, region, and ethnicity). Univariate and multivariate logistic regression models were applied to identify factors associated with astigmatism prevalence and correction status, calculating odds ratios (OR) and their corresponding 95% confidence intervals (CI). A *P*-value < 0.05 was considered statistically significant.

## RESULTS

### Basic information and refractive status

**Figure 1:**
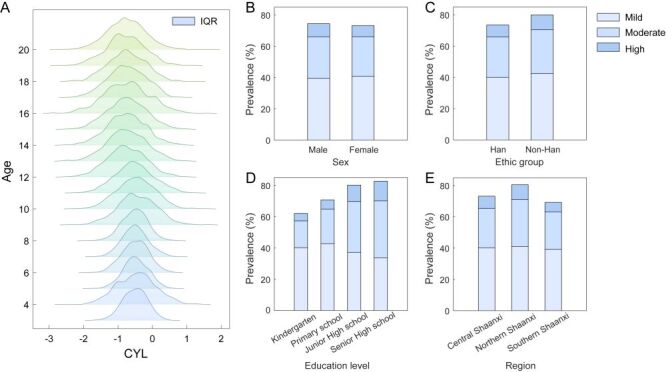
Distribution of astigmatism severity across demographic groups. **Panel A.** Distribution of CYL across age groups. The x-axis shows astigmatism magnitude, the y-axis indicates age, and box plots represent IQR. **Panels B–E.** Astigmatism prevalence (%) by different demographic factors: **Panel B.** Sex, **Panel C.** Ethnicity, **Panel D.** Education level, and **Panel E.** Geographic region. Stacked bars in B–E are color-coded to represent different astigmatism severity levels: light blue for mild astigmatism (0.50 ≤ CYL < 1.00 D), medium blue for moderate astigmatism (1.00 ≤ CYL < 2.00 D), and dark blue for high astigmatism (CYL ≥ 2.00 D). CYL – cylindrical dioptre, IQR – interquartile range.

A total of 236 397 children and adolescents (51.71% male) were included in the analysis, with a median age of 11.0 years (IQR = 8.5, 14.0) years. The median uncorrected visual acuity was 0.1 logarithm of the minimum angle of resolution (logMAR) (IQR = 0, 0.6), a logarithmic scale for visual acuity in which 0.0 logMAR corresponds to 20/20 vision. The refractive profile of the cohort was characterised by a median spherical dioptre of −0.5 D (IQR = −2.25, 0.25), a CYL of −0.5 D (IQR = −1.0, −0.25), and a SE of −0.875 D (IQR = −2.625, 0) (Table S1 in the **Online Supplementary Document**). All these parameters varied significantly with demographic factors (all *P* < 0.05). Notably, CYL exhibited a progressive decline with increasing age (**Figure 1**, Panel A).

### Astigmatism prevalence and severity analysis

The overall prevalence of astigmatism (CYL≥0.5 D) was 73.81%, increasing significantly with age from 63.17% (3–5 years) to 81.7% (18–20 years) and education level, with the highest prevalence (82.26%) observed in the 15–17 years age group (**Table 1**). Consequently, when applying the more stringent threshold of CYL ≥ 1D, astigmatism prevalence was 45.61%. Males exhibited a higher prevalence of astigmatism (74.44%) compared to females (73.13%), and ethnic minorities showed a higher prevalence (79.89%) than the Han population (73.47%). Geographic variation was substantial, with Northern Shaanxi (80.52%) showing the highest prevalence, followed by Central (73.18%) and Southern regions (69.21%) (Figure S1 in the **Online Supplementary Document**).

**Table 1:** Prevalence and severity distribution of astigmatism across different demographic groups

Group	N	Astigmatism n (%)	Mild astigmatism n (%)	Moderate astigmatism n (%)	High astigmatism n (%)	χ2	*P*-value
**Age (year)**							
3–5	4694	2965 (63.17)	1901 (64.11)	844 (28.47)	220 (7.42)	5937.58	<0.001
6–8	66 895	43 476 (64.99)	28 541 (65.65)	11 687 (26.88)	3248 (7.47)		
9–11	66 632	48 793 (73.23)	28 620 (58.66)	16 077 (32.95)	4096 (8.39)		
12–14	54 977	43 739 (79.56)	21 052 (48.13)	17 384 (39.74)	5303 (12.12)		
15–17	37 456	30 812 (82.26)	12 719 (41.28)	13 514 (43.86)	4579 (14.86)		
18–20	5743	4692 (81.70)	2059 (43.88)	1894 (40.37)	739 (15.75)		
**Education level**							
Kindergarten	15 847	9838 (62.08)	6370 (64.75)	2707 (27.52)	761 (7.74)	5220.03	<0.001
Primary school	137 298	97 044 (70.68)	58 738 (60.53)	30 311 (31.23)	7995 (8.24)		
Junior high school	50 034	40 119 (80.18)	18 592 (46.34)	16 296 (40.62)	5231 (13.04)		
Senior high school	33 218	27 476 (82.71)	11 192 (40.73)	12 086 (43.99)	4198 (15.28)		
**Sex**							
Male	122 248	90 996 (74.44)	48 280 (53.06)	32 518 (35.74)	10 198 (11.21)	190.13	<0.001
Female	114 149	83 481 (73.13)	46 612 (55.84)	28 882 (34.60)	7987 (9.57)		
**Region**							
Central Shaanxi	120 319	88 049 (73.18)	48 387 (54.95)	30 283 (34.39)	9379 (10.65)	413.65	<0.001
Northern Shaanxi	53 834	43 348 (80.52)	22 090 (50.96)	16 192 (37.35)	5066 (11.69)		
Southern Shaanxi	62 244	43 080 (69.21)	24 415 (56.67)	14 925 (34.64)	3740 (8.68)		
**Ethic group**							
Han	224 086	164 642 (73.47)	89 675 (54.47)	57 946 (35.20)	17 021 (10.34)	23.42	<0.001
Non-Han	12 311	9835 (79.89)	5217 (53.05)	3454 (35.12)	1164 (11.84)		
All	236 397	174 477 (73.81)	94 892 (54.39)	61 400 (35.19)	18 185 (10.42)		

Regarding severity distribution, low astigmatism predominated overall (54.39%), while moderate-to-high astigmatism increased markedly with age and education level. Males had a lower proportion of low astigmatism but higher proportions of moderate and high astigmatism compared to females. Northern Shaanxi exhibited the most severe profile, with only 50.96% having low astigmatism. All demographic and geographic comparisons showed statistically significant differences in severity distribution (all *P* < 0.001, **Table 1**).

### Astigmatism type analysis

**Figure 2:**
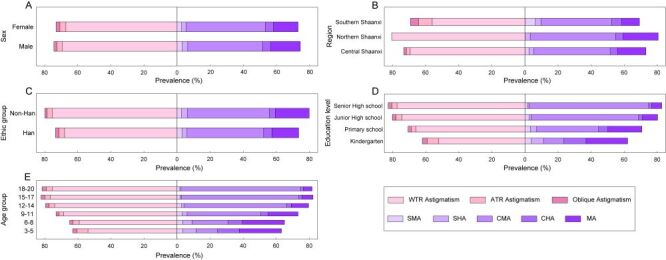
Distribution of astigmatism types across demographic groups. **Panel A.** Sex, **Panel B.** Region, **Panel C.** Ethnicity, **Panel D.** Education level, **Panel E.** Age group. The x-axis represents astigmatism prevalence, and the y-axis represents different demographic categories. The pink section illustrates astigmatism by-axis orientation, while the purple section represents astigmatism types based on the relationship between the two principal meridians and the retina. CHA – compound hyperopic astigmatism, CMA – compound myopic astigmatism, MA – mixed astigmatism, SHA – simple hyperopic astigmatism, SMA – simple myopic astigmatism.

Based on axis orientation, with-the-rule astigmatism (WTR) predominated (92.63%), followed by against-the-rule (ATR) astigmatism (4.55%) and oblique astigmatism (2.82%). WTR proportion increased with age, peaking at 93.74% in the 9–11 years group, while ATR and oblique astigmatism decreased correspondingly (**Figure 2**; Table S2 in the **Online Supplementary Document**). Notably, all astigmatic children in Northern Shaanxi exhibited WTR. Gender, regional, and ethnic differences were statistically significant (all *P* < 0.001).

Based on the relationship between the two principal meridians and the retina, compound myopic astigmatism (CMA) was most common (63.00%), followed by mixed astigmatism (MA) (22.22%), compound hyperopic astigmatism (CHA) (6.67%), simple hyperopic astigmatism (SHA) (4.19%), and simple myopic astigmatism (SMA) (3.92%). In the present study, CMA increased markedly with age and education level, while other types decreased (**Figure 2**; Table S3 in the **Online Supplementary Document**). Males had higher proportions of SMA, SHA, and MA compared to females. All demographic comparisons showed significant differences (all *P* < 0.001).

Analysis across severity levels confirmed that the hierarchy of axis orientation (WTR > ATR > oblique) remained consistent. However, the distribution of refractive types varied with severity, with SHA being more common than SMA only in the low astigmatism group (Table S4 in the **Online Supplementary Document**). Both axis and type distributions differed significantly across severity levels (both *P* < 0.001).

### Analysis of factors affecting astigmatism

Astigmatism risk increased with age, peaking in the 15–17 years group (univariate OR = 2.704; 95% CI = 2.534, 2.886; adjusted OR = 1.786; 95% CI = 1.618, 1.97, both *P* < 0.001). Similarly, risk increased with education levels, with high school students showing the highest risk (univariate OR = 2.923; 95% CI = 2.800, 3.051; adjusted OR = 1.617; 95% CI = 1.487, 1.758, both *P* < 0.001). Females exhibited a protective effect *vs*. males in both analyses (OR = 0.935; 95% CI = 0.917, 0.952, *P* < 0.001). Geographically, Northern Shaanxi showed increased risk (adjusted OR = 1.452; 95% CI = 1.416, 1.49), while Southern Shaanxi showed decreased risk (adjusted OR = 0.807; 95% CI = 0.79, 0.825) compared to Central Shaanxi (both *P* < 0.001). Ethnic minorities had higher risk than the Han population, which remained significant after adjustment (OR = 1.319; 95% CI = 1.259, 1.381, *P* < 0.001). Effect estimates were attenuated after adjustment, but all associations remained significant, confirming these as independent risk factors (**Table 2**).

**Table 2:** Associations between the prevalence of astigmatism and associated factors

Variables	Univariate logistic regression analysis			Multivariate logistic regression analysis*		
	**OR**	**95% CI**	***P*-value**	**OR**	**95% CI**	***P*-value**
**Age (year)**						
3–5	reference			reference		
6–8	1.083	1.018, 1.151	0.011	0.935	0.871, 1.004	0.063
9–11	1.595	1.499, 1.697	<0.001	1.326	1.23, 1.429	<0.001
12–14	2.270	2.131, 2.417	<0.001	1.723	1.586, 1.872	<0.001
15–17	2.704	2.534, 2.886	<0.001	1.786	1.618, 1.97	<0.001
18–20	2.603	2.381, 2.847	<0.001	1.622	1.435, 1.834	<0.001
**Education level**						
Kindergarten	reference			reference		
Primary school	1.472	1.423, 1.524	<0.001	1.204	1.154, 1.256	<0.001
Junior high school	2.471	2.377, 2.569	<0.001	1.373	1.29, 1.462	<0.001
Senior high school	2.923	2.8, 3.051	<0.001	1.617	1.487, 1.758	<0.001
**Sex**						
Male	reference			reference		
Female	0.935	0.918, 0.952	<0.001	0.935	0.917, 0.952	<0.001
**Region**						
Central Shaanxi	reference			reference		
Northern Shaanxi	1.515	1.478, 1.553	<0.001	1.452	1.416, 1.49	<0.001
Southern Shaanxi	0.824	0.807, 0.842	<0.001	0.807	0.79, 0.825	<0.001
**Ethic group**						
Han	reference			reference		
Non-Han	1.434	1.371, 1.5	<0.001	1.319	1.259, 1.381	<0.001

CI – confidence interval, OR – odds ratio

*The multivariable models were adjusted for age, education level (kindergarten to senior high school) sex, region (Central Shaanxi, northern Shaanxi, and southern Shaanxi), and ethnic group.

### Analysis of astigmatism correction

**Figure 3:**
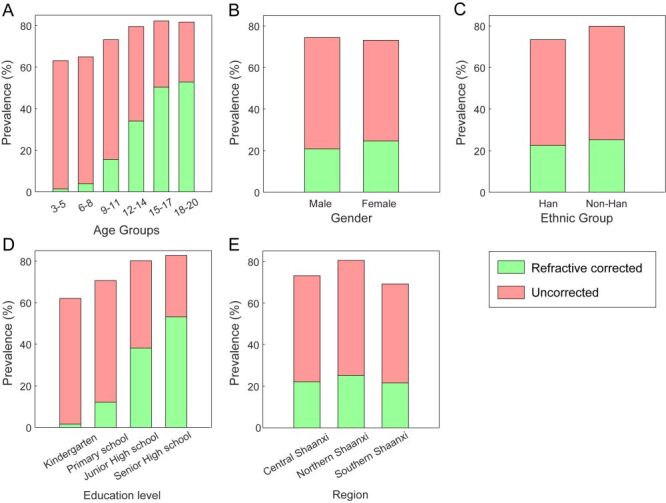
Analysis of astigmatism correction rates across different demographic groups. **Panel A.** Age group. **Panel B.** Sex. **Panel C.** Ethnicity. **Panel D.** Education level. **Panel E.** Region. The y-axis shows astigmatism prevalence (%). Green segments represent the proportion of individuals with astigmatism who received refractive correction, while red segments represent those who remained uncorrected.

Overall, 30.73% of subjects with astigmatism had received refractive correction. Correction rates increased with age and education level. Females had higher correction rates than males (33.70 *vs*. 28.00%) (*P* < 0.001). Regional differences were modest, with Northern Shaanxi showing slightly higher rate (31.20%) (*P* < 0.001). Detailed correction patterns by demographic groups are presented in **Figure 3** and Table S5 in the **Online Supplementary Document**.

Multivariate logistic regression identified multiple factors independently associated with correction status (**Table 3**). Astigmatism severity was the strongest predictor, with moderate (adjusted OR = 2.365; 95% CI = 2.302, 2.430) and high astigmatism (adjusted OR = 6.155; 95% CI = 5.896, 6.425) showing substantially higher correction likelihood compared to mild astigmatism (both *P* < 0.001). Older age and higher education levels were associated with increased correction rates, with senior high school students having the highest likelihood (adjusted OR = 4.402; 95% CI = 3.699, 5.238, *P* < 0.001). Females were more likely to receive correction than males (adjusted OR = 1.405; 95% CI = 1.370, 1.441, *P* < 0.001). For astigmatism type, CMA had markedly higher correction rates (adjusted OR = 15.365; 95% CI = 13.549, 17.42, *P* < 0.001) compared to simple myopic astigmatism, while against-the-rule astigmatism showed lower correction rates than with-the-rule type (adjusted OR = 0.713; 95% CI = 0.666, 0.763, *P* < 0.001).

**Table 3:** Associations between astigmatism correction rates and associated factors

Variables	Univariate logistic regression analysis			Multivariate logistic regression analysis*		
	**OR**	**95% CI**	***P*-value**	**OR**	**95% CI**	***P*-value**
**Age (year)**						
3–5	reference			reference		
6–8	2.847	2.220, 3.652	<0.001	1.245	0.928, 1.670	0.144
9–11	12.062	9.424, 15.438	<0.001	3.220	2.391, 4.337	<0.001
12–14	33.258	25.991, 42.557	<0.001	5.050	3.742, 6.816	<0.001
15–17	70.248	54.881, 89.919	<0.001	6.516	4.807, 8.834	<0.001
18–20	81.642	63.393, 105.145	<0.001	6.867	5.024, 9.386	<0.001
**Education level**						
Kindergarten	reference			reference		
Primary school	8.048	7.086, 9.142	<0.001	1.901	1.623, 2.226	<0.001
Junior high school	35.179	30.958, 39.976	<0.001	2.955	2.505, 3.487	<0.001
Senior high school	69.605	61.200, 79.164	<0.001	4.402	3.699, 5.238	<0.001
**Sex**						
Male	reference			reference		
Female	1.307	1.280, 1.334	<0.001	1.405	1.370, 1.441	<0.001
**Region**						
Central Shaanxi	reference			reference		
Northern Shaanxi	1.044	1.018, 1.070	0.001	0.873	0.846, 0.901	<0.001
Southern Shaanxi	1.041	1.015, 1.067	0.002	1.070	1.036, 1.104	<0.001
**Ethic groups**						
Han	reference			reference		
Non-Han	1.045	1.000, 1.092	0.048	1.402	1.327, 1.481	<0.001
**Astigmatism severity**						
Mild	reference			reference		
Moderate	2.610	2.551, 2.669	<0.001	2.365	2.302, 2.430	<0.001
High	4.964	4.802, 5.132	<0.001	6.155	5.896, 6.425	<0.001
**Astigmatism type based on axis orientation**						
WTR	reference			reference		
ATR	0.558	0.528, 0.590	<0.001	0.713	0.666, 0.763	<0.001
Oblique astigmatism	0.757	0.710, 0.808	<0.001	0.916	0.848, 0.990	0.027
**Astigmatism type based on refraction**						
SMA	reference			reference		
SHA	0.521	0.427, 0.636	<0.001	0.991	0.808, 1.216	0.933
CMA	19.633	17.395, 22.158	<0.001	15.365	13.549, 17.424	<0.001
CHA	2.781	2.432, 3.181	<0.001	4.079	3.542, 4.697	<0.001
MA	1.456	1.281, 1.654	<0.001	1.527	1.336, 1.744	<0.001

ATR – against-the-rule, CHA – compound hyperopic astigmatism, CI – confidence interval, CMA – compound myopic astigmatism, MA – mixed astigmatism, OR – odds ratio, SHA – simple hyperopic astigmatism, SMA – simple myopic astigmatism, WTR – with-the-rule

*The multivariable models were mutually adjusted for age, education level, sex, region, ethnic group, astigmatism severity, and astigmatism type (axis and refraction).

## DISCUSSION

This large-scale study investigated 236 397 children and adolescents aged 3–20 years in Shaanxi Province and found an overall astigmatism prevalence of 73.81%, with low astigmatism, with-the-rule astigmatism, and compound myopic astigmatism being the predominant types. Further analysis revealed that age, education level, gender, region, and ethnicity significantly influenced astigmatism prevalence. Notably, despite the high prevalence, only 30.73% of children and adolescents with astigmatism received refractive correction, highlighting a concerning gap that warrants urgent attention from clinical and public health perspectives.

Numerous cross-sectional and longitudinal studies have previously reported astigmatism prevalence among Chinese children and adolescents [9,12–24,26,39,40], with specific prevalence including 26.11% among 3–7-year-olds in Guangxi [9], 36% in Wuxi [26], 46.1% among 3–7-year-olds in Beijing [14], 21.4% among 6–8-year-olds in Hong Kong [15], 59.3% in Xi'an [16], 57.3% in Tianjin [12], 36.1% in Xinjiang [13], 46.07% in Dalian [21], and 26.2% in Qinghai [39]. Even when recalculated using the threshold of CYL ≥ 1.00 D, our prevalence rate of 45.61% remains significantly higher than reports from other regions using the same criteria.

The observed geographic variation within Shaanxi Province itself – with a clear south-to-north increasing gradient peaking at 84.05% in Yulin City (**Figure 2**) – further underscores the potential influence of environmental and demographic factors. Our study identified significant regional variations within Shaanxi Province, with prevalence of 73.18% in Central Shaanxi, 80.52% in Northern Shaanxi, and 69.21% in Southern Shaanxi. Multivariate analysis confirmed that Northern Shaanxi residence independently increased astigmatism risk (OR = 1.452; 95% CI = 1.416, 1.49), while Southern Shaanxi showed a protective effect (OR = 0.807; 95% CI = 0.790, 0.825) compared to Central regions. These significant regional differences may be attributed to variations in ethnic composition, living environments, and lifestyle habits across different geographical areas. It is important to note that our study employed a relatively stringent threshold for astigmatism definition (|CYL| ≥ 0.5 D), which may partially explain the higher prevalence observed, though this threshold is consistent with several previous studies [12,14,16,19,41]. In contrast, other studies adopted different diagnostic criteria, with some defining astigmatism at 0.75 D [13,17,21,39], others at 1.00 D [9,15,26], and some at 1.50 D [20,40].

Our data demonstrate that astigmatism prevalence increases significantly with age and education level, rising from 63.17% in 3–5-year-olds to 81.70% in 18–20-year-olds. The prevalence in kindergarten, primary school, junior high school, and senior high school were 62.08, 70.68, 80.18, and 82.71%, respectively, which is consistent with most previous studies [13,16–19,26,40]. In multivariate regression analysis, after adjusting for other factors, the odds of astigmatism peaked in the 15–17 years group (OR = 1.786; 95% CI = 1.618, 1.97), while education level showed a positive correlation with astigmatism risk, with high school students having 1.617 times higher odds compared to kindergarten children.

Males exhibited both higher prevalence (74.44 *vs*. 73.13%) and greater severity of astigmatism compared to females, which is consistent with many previous studies [13,16,41]. After adjustment, males had approximately 1.07 times higher odds of astigmatism than females. This result suggests that males may be a relatively high-risk population for astigmatism development, though the literature shows mixed findings, with some studies reporting no gender differences [40] or higher prevalence in girls [24]. These gender differences require further exploration in future research. Additionally, the astigmatism prevalence in ethnic minority populations (79.89%) was higher than in the Han population (73.47%), which contradicts the findings of other studies [13,17] and may reflect our sample's unbalanced ethnic composition.

Our analysis of astigmatism severity shows that mild astigmatism (0.50–1.00 D) accounted for the largest proportion (54.39%), followed by moderate (35.19%) and high astigmatism (10.42%), consistent with Wang et al [19]. As age and education increased, the proportion of moderate and high astigmatism increased. Males had higher proportions of moderate and high astigmatism than females, while Northern Shaanxi had the lowest proportion of low-grade astigmatism (50.96%), aligning with the highest overall prevalence observed in that region.

The clinical significance of these patterns is substantial, as eyes with high astigmatism exhibit faster axial elongation [12] and astigmatism is associated with both myopia progression [10–13] and compromised visual function [3–5,8,9]. Although mild astigmatism was most common in our cohort, the 45.61% proportion of moderate-to-high astigmatism underscores the need for targeted clinical management. Clinicians should provide regular monitoring for children with mild astigmatism, implement timely optical correction for those with moderate-to-high astigmatism, and conduct further corneal assessments in children with high astigmatism to screen for keratoconus and related corneal diseases.

Based on the classification by the positions of the two principal meridians relative to the retina, CMA was most common (63.00%), followed by MA (22.22%), CHA (6.67%), SHA (4.19%), and SMA (3.92%). This distribution differs from the findings of Shah et al [29] in children aged 3–11 years in Islamabad, where MA was most prevalent. The proportion of CMA increased with both age and educational level, consistent with Shen et al [42] and Wang et al [19], suggesting an age-related rise in myopic astigmatism. The trend may reflect the physiological course of myopia development during adolescence and the concurrent increase in near-work demands with higher grades. When categorised by-axis, WTR astigmatism was predominant (92.63%), followed by ATR (4.55%) and oblique astigmatism (2.82%), consistent with prior reports [12,13,19,20,24,26,43]. The proportion of WTR peaked at 9–11 years (93.74%), while ATR and oblique astigmatism declined with age.

Regional variations were particularly striking, with Northern Shaanxi displaying an exceptional pattern where 100% of astigmatic children had WTR astigmatism. The refractive profiles also varied considerably by region, with Southern Shaanxi showing higher SMA and CHA rates while Northern Shaanxi had virtually no SMA but the highest MA prevalence. These pronounced regional differences likely reflect complex interactions between environmental exposures, lifestyle factors, and potentially genetic backgrounds specific to each geographical area. Our analysis also revealed that as astigmatism severity increased, WTR proportion rose while ATR and oblique astigmatism declined. Similarly, SMA and SHA decreased while MA increased. These patterns align with previous research suggesting that different astigmatism types may influence refractive development differently, with some studies indicating faster axial elongation in ATR [12] and potential myopia suppression through choroidal thickening in WTR [44]. Although ATR was not the predominant type in our population, affected children require careful monitoring given the potential implications for myopia progression.

Perhaps most concerning from a public health perspective is that only 30.73% of children with astigmatism received refractive correction. This proportion increased significantly with age, from just 2.19% in the 3–5-year group to 64.66% in the 18–20-year group, and similarly with education level (2.51% in kindergarten to 64.19% in high school). This trend likely reflects enhanced awareness of vision problems and increased demand for visual correction due to greater academic requirements. Females had significantly higher correction rates than males (33.70 *vs*. 28.00%), while regional differences were minimal. Correction rates for high astigmatism were consistently higher than for low and moderate astigmatism, rising to 77.00% in the 18–20-year group. However, overall correction rates remained low, particularly among younger children and those with lower education levels, highlighting the need for enhanced astigmatism screening and intervention, especially for younger populations. Uncorrected astigmatism creates varying degrees of visual blur due to different defocus amounts across meridians [6]. Research has established that individuals with astigmatism exhibit compromised visual capabilities across multiple domains [3,5,6,8,9]. These impairments in visual function not only affect children's daily activities and learning but, more seriously, early uncorrected astigmatism may place children at substantial risk for amblyopia [6,7]. Multiple studies have shown that early optical correction can prevent the impact of astigmatism on visual function [45–47], emphasising the importance of timely intervention.

### Study strengths and limitations

Our study provides a comprehensive analysis of astigmatism in Shaanxi Province. However, several limitations warrant consideration. First, the cross-sectional design precludes the determination of causal relationships or natural progression of astigmatism over time. Second, we did not collect detailed information on lifestyle factors such as visual habits, reading behaviours, electronic device usage, sleep patterns, physical activity levels, study habits, or family history, limiting our ability to analyse their potential influences on astigmatism development. Third, our threshold for astigmatism definition (|CYL| ≥ 0.5 D) is relatively low, potentially resulting in higher prevalence estimates than studies using more stringent criteria, necessitating caution when making direct comparisons. Fourth, although analysing only the eye with the higher absolute cylindrical value can reflect the maximum refractive burden, it may still lead to an overestimation of astigmatism prevalence. Finally, cycloplegic examinations were not performed on all participants; although previous research [48,49] suggests minimal impact on cylindrical values, more comprehensive investigations are needed in future studies.

## CONCLUSIONS

This investigation reveals important patterns in astigmatism prevalence, type distribution, influencing factors, and correction status among 3–20-year-olds in Shaanxi Province. The prevalence increased with age and education level, was higher in males than in females, higher in Northern Shaanxi compared to Central and Southern regions, and higher among ethnic minorities than in the Han population. With-the-rule astigmatism and compound myopic astigmatism were the most prevalent types, while correction rates remained suboptimal, particularly among younger children.

These findings emphasise the importance of implementing early astigmatism screening and timely intervention programmes for children and adolescents, while also highlighting the need for further research into the effects of regional, ethnic, behavioural, and lifestyle factors on astigmatism development to formulate targeted prevention and management strategies. From a public health perspective, policymakers should strengthen school-based vision screening and ensure equitable access to affordable refractive services, particularly in rural and minority regions. Clinicians and educators can collaborate to improve awareness among parents and teachers regarding early signs of visual problems and encourage prompt eye examinations, to reduce the long-term educational and developmental impact of uncorrected refractive errors.

## Additional material


Online Supplementary Document
