# Supplementary material for: Characterisation and risk factors of astigmatism among children and adolescents aged 3–20 years in Northwestern China
Source: J Glob Health. 2026 Feb 6;16:04047. doi: 10.7189/jogh.16.04047 (PMC12879261; doi:10.7189/jogh.16.04047)
Supplement: Online Supplementary Document [file jogh-16-04047-s001.pdf]

Supplement to: Wei JH, Guo YM, Wang JQ, Han YJ, Yao Q, Zhang GY, Ye L. Characterisation and risk factors of astigmatism among children and adolescents aged 3–20 years in Northwestern China. J Glob Health. 2026;16:04047.

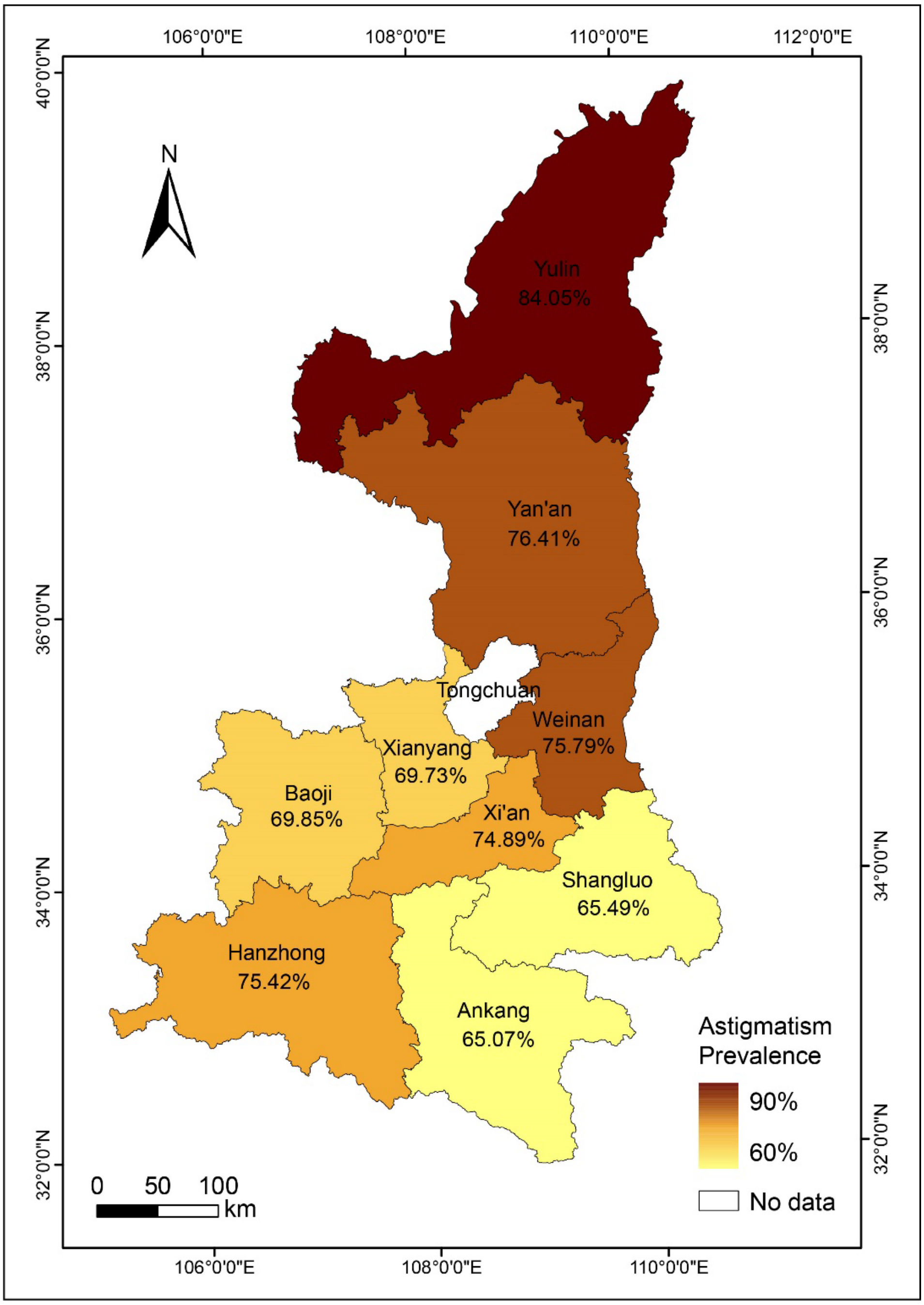

Figure S1. Geographic distribution of astigmatism prevalence across cities in Shaanxi Province

Table S1. Demographic Characteristics, Visual Acuity, and Refractive Parameters

Across Different Groups

Group	N (%)	LogMAR VA*	DS*	CYL*	SE*
Age (year)					
3-5	4,694 (1.99%)	0.1 (0,0.2)	0.25 (0,0.75)	-0.5 (-0.75, -0.25)	0.125 (-0.375,0.5)
6-8	66,895 (28.30%)	0 (0,0.1)	0 (-0.5,0.5)	-0.5 (-0.75, -0.25)	-0.125 (-0.75,0.375)
9-11	66,632 (28.19%)	0.1 (0,0.4)	-0.5 (-1.5,0.25)	-0.5 (-1, -0.25)	-0.75 (-2, -0.125)
12-14	54,977 (23.26%)	0.4 (0,0.8)	-1.5 (-3, -0.25)	-0.75 (-1.25, -0.5)	-1.75 (-3.5, -0.625)
15-17	37,456 (15.84%)	0.6 (0.2,0.9)	-2.5 (-4, -0.75)	-0.75 (-1.25, -0.5)	-2.875 (-4.5, -1.25)
18-20	5,743 (2.43%)	0.7 (0.2,1.0)	-2.75 (-4.5, -1.25)	-0.75 (-1.25, -0.5)	-3.25 (-5, -1.5)
H		39943.11	59242.83	14349.19	63109.83
P		<0.001	<0.001	<0.001	<0.001
Education level					
Kindergarten	15,847 (6.70%)	0.1 (0,0.2)	0.25 (0,0.75)	-0.5 (-0.75, -0.25)	0.125 (-0.375, 0.5)
Primary school	137,298 (58.08%)	0.0 (0,0.3)	-0.25 (-1.25,0.25)	-0.5 (-1, -0.25)	-0.5 (-1.625, 0)
Junior High school	50,034 (21.17%)	0.4 (0.1,0.8)	-1.75 (-3.25, -0.5)	-0.75 (-1.25, -0.5)	-2.125 (-3.75, -0.75)
Senior High school	33,218 (14.05%)	0.6 (0.2,1.0)	-2.5 (-4.25, -1)	-0.75 (-1.25, -0.5)	-3.12 (-4.75, -1.375)
H		34260.28	50400.23	11859.13	53358.71
P		<0.001	<0.001	<0.001	<0.001
Sex					
Male	122,248 (51.71%)	0.1 (0,0.5)	-0.5 (-2,0.25)	-0.5 (-1, -0.25)	-0.75 (-2.375,0)
Female	114,149 (48.29%)	0.2 (0,0.6)	-0.75 (-2.25,0.12)	-0.5 (-1, -0.25)	-1 (-2.75, -0.125)
Z		44.17	27.00	12.30	22.69
P		<0.001	<0.001	<0.001	<0.001
Region					
Central Shaanxi	120,319 (50.90%)	0.1 (0,0.5)	-0.5 (-2,0.25)	-0.5 (-1, -0.25)	-0.875 (-2.5, -0.06)
Northern Shaanxi	53,834 (22.77%)	0.2 (0,0.7)	-0.75 (-2.5,0.25)	-0.75 (-1.25, -0.5)	-1.125 (-3, -0.005)
Southern Shaanxi	62,244 (26.33%)	0.1 (0,0.6)	-0.5 (-2,0.25)	-0.5(-1, -0.25)	-0.75 (-2.375,0)
H		2129.26	252.21	1609.94	427.39
P		<0.001	<0.001	<0.001	<0.001
Ethic groups					
Han	224,086 (94.79%)	0.1 (0,0.5)	-0.5 (-2.25,0.25)	-0.5 (-1, -0.25)	-0.875 (-2.625,0)
Non-Han	12,311 (5.21%)	0.2 (0,0.6)	-0.5 (-2.25,0.25)	-0.75 (-1, -0.25)	-1(-2.625,0)
Z		13.80	1.25	6.72	2.38
P		<0.001	0.212	<0.001	0.018

VA – visual acuity, DS – degree of sphere, CYL – cylindrical diopter, SE – sphere equivalent.

*Data are expressed as median (IQR).

Table S2. Distribution of Astigmatism Axis Orientation Types Across Different Demographic Groups

Group	Astigmatism (N)	WTR Astigmatism [N (%)]	ATR Astigmatism [N (%)]	Oblique Astigmatism [N (%)]	χ2	P
Age (year)						
3-5	2,965	2,530(85.33%)	310(10.46%)	125(4.22%)	583.55	<0.001
6-8	43,476	39,593(91.07%)	2,511(5.78%)	1,372(3.16%)		
9-11	48,793	45,737(93.74%)	1,822(3.73%)	1,234(2.53%)		
12-14	43,739	40,703(93.06%)	1,841(4.21%)	1,195(2.73%)		
15-17	30,812	28,718(93.20%)	1,251(4.06%)	843(2.74%)		
18-20	4,692	4,333(92.35%)	208(4.43%)	151(3.22%)		
Education level						
Kindergarten	9,838	8,260(83.96%)	1,112(11.30%)	466(4.74%)	1281.10	<0.001
Primary school	97,044	90,431(93.19%)	4,016(4.14%)	2,597(2.68%)		
Junior High school	40,119	37,223(92.78%)	1,774(4.42%)	1,122(2.80%)		
Senior High school	27,476	25,700(93.54%)	1,041(3.79%)	735(2.68%)		
Sex						
Male	90,996	84,944(93.35%)	3,803(4.18%)	2,249(2.47%)	150.69	<0.001
Female	83,481	76,670(91.84%)	4,140(4.96%)	2,671(3.20%)		
Region						
Central Shaanxi	88,049	83,408(94.73%)	2,809(3.19%)	1,832(2.08%)	12681.04	<0.001
Northern Shaanxi	43,348	43,348(100.00%)	0(0.00%)	0(0.00%)		
Southern Shaanxi	43,080	34,858(80.91%)	5,134(11.92%)	3,088(7.17%)		
Ethic group						
Han	164,642	152,328(92.52%)	7,565(4.59%)	4,749(2.88%)	58.32	<0.001
Non-Han	9,835	9,286(94.42%)	378(3.84%)	171(1.74%)		
All	174,477	161,614(92.63%)	7,943(4.55%)	4,920(2.82%)		

WTR – With-the-Rule, ATR – Against-the-Rule.

Table S3. Distribution of astigmatism types across different demographic groups

Group	Astigmatism (N)	SMA [N (%)]	SHA [N (%)]	CMA [N (%)]	CHA [N (%)]	MA [N (%)]	χ2	P
Age (year)								
3-5	2,965	168 (5.67 %)	375 (12.65%)	610 (20.57%)	615 (20.74%)	1,197 (40.37%)	32859.9 4	<0.001
6-8	43,476	2,311 (5.32 %)	3,785 (8.71%)	14,577 (33.53%)	5,793 (13.32%)	17,010 (39.13%)		
9-11	48,793	2,107 (4.32 %)	1,975 (4.05%)	29,748 (60.97%)	2,932 (6.01%)	12,031 (24.66%)		
12-14	43,739	1478	829	34,206	1,461	5,765		

		(3.38 %)	(1.90%)	(78.20%)	(3.34%)	(13.18%)		
15-17	30,812	671 (2.18 %)	316 (1.03%)	26,613 (86.37%)	744 (2.41%)	2,468 (8.01%)		
18-20	4,692	100 (2.13 %)	39 (0.83%)	4,161 (88.68%)	90 (1.92%)	302 (6.44%)		
Education level								
Kindergarten	9,838	614 (6.24 %)	1,163 (11.82%)	1,957 (19.89%)	2,116 (21.51%)	3,988 (40.54%)	26401.38	<0.001
Primary school	97,044	4,434 (4.57 %)	5,302 (5.46%)	51,383 (52.95%)	7,724 (7.96%)	28,201 (29.06%)		
Junior High school	40,119	1,254 (3.13 %)	599 1.49%)	32,512 (81.04%)	1,187 (2.96%)	4,567 (11.38%)		
Senior High school	27,476	533 (1.94 %)	255 (0.93%)	24,063 (87.58%)	608 (2.21%)	2,017 (7.34%)		
Sex								
Male	90,996	3,743 (4.11 %)	4,065 (4.47%)	55,399 (60.88%)	5,972 (6.56%)	21,817 (23.98%)	453.76	<0.001
Female	83,481	3,092 (3.70 %)	3,254 (3.90%)	54,516 (65.30%)	5,663 (6.78%)	16,956 (20.31%)		
Region								
Central Shaanxi	88,049	2,917 (3.31 %)	3,543 (4.02%)	55,479 (63.01%)	5,350 (6.08%)	20,760 (23.58%)	6483.07	<0.001
Northern Shaanxi	43,348	5(0.01 %)	2,071 (3.93%)	26,633 (64.14%)	3,822 (5.68%)	6,641 (26.23%)		
Southern Shaanxi	43,080	3,913 (9.08 %)	7,319 (4.81%)	109,915 (61.82%)	11,635(8.87%)	38,773 (15.42%)		
Ethic group								
Han	164,642	6,517 (3.96 %)	6,867 (4.17%)	103,812 (63.05%)	11,203 (6.80%)	36,243 (22.01%)	156.40	<0.001
Non-Han	9,835	318 (3.23 %)	452 (4.60%)	6,103 (62.05%)	432 (4.39%)	2,530 (25.72%)		
All	174,477	6,835 (3.92 %)	7,319 (4.19%)	109,915 (63.00%)	11,635 (6.67%)	38,773 (22.22%)		

SMA – Simple Myopic Astigmatism, SHA – Simple Hyperopic Astigmatism, CMA – Compound Myopic Astigmatism, CHA – Compound Hyperopic Astigmatism, MA – Mixed Astigmatism.

Table S4. Distribution of Astigmatism Types Across Different Degrees of Astigmatism

Severity

Group	N	WTR Astigmatism [N (%)]	ATR Astigmatism [N (%)]	Oblique Astigmatism [N (%)]	χ2	P	SMA [N (%)]	SHA [N (%)]	CMA [N (%)]	CHA [N (%)]	MA [N (%)]	χ2	P
Mild	94,892	85,267 (89.86%)	5,756 (6.07%)	3,869 (4.08%)	2398.72	<0.001	4,580 (4.83%)	5,743 (6.05%)	57,338 (60.42%)	7,516 (7.92%)	19,715 (20.78%)	3245.47	<0.001
Moderate	61,400	58,790 (95.75%)	437 (2.85%)	191 (1.40%)			1,883 (3.07%)	184 (2.27%)	11,754 (66.49%)	982 (5.11%)	4,893 (23.07%)		
High	18,185	17,557 (96.55%)	7,943 (2.40%)	4,920 (1.05%)			372 (2.05%)	7,319 (1.01%)	109,915(64.64%)	11,635(5.40%)	38,773 (26.91%)		
All	174,477	161,614 (92.63%)	7,943 (4.55%)	4,920 (2.82%)			6,835 (3.92%)	7,319 (4.19%)	109,915(63.00%)	11,635(6.67%)	38,773(22.22%)		

WTR – With-the-Rule, ATR – Against-the-Rule, SMA – Simple Myopic Astigmatism, SHA – Simple Hyperopic Astigmatism, CMA – Compound Myopic Astigmatism, CHA – Compound Hyperopic Astigmatism, MA – Mixed Astigmatism.

Table S5. Analysis of astigmatism correction status among different population groups in children and adolescents in Shaanxi Province.

Group	Astigmatism corrected [N (%)]	Astigmatism uncorrected [N (%)]	χ2	P	Mild astigmatism corrected [N (%)]	Moderate astigmatism corrected [N (%)]	High astigmatism corrected [N (%)]	χ2	P
Age (year)									
3-5	65(2.19%)	2,900(97.81%)	34561.01	<0.001	11(0.58%)	21(2.49%)	33(15.00%)	499.001	<0.001
6-8	2,608(6.00%)	40,868(94.00%)			826(2.89%)	908(7.77%)	874(26.91%)		
9-11	10,384(21.28%)	38,409(78.72%)			3,955(13.82%)	4,525(28.15%)	1,904(46.48%)		
12-14	18,680(42.71%)	25,059(57.29%)			6,699(31.82%)	8,633(49.66%)	3,348(63.13%)		
15-17	18,844(61.16%)	11,968(38.84%)			6,481(50.96%)	8,978(66.43%)	3,385(73.92%)		
18-20	3,034(64.66%)	1,658(35.34%)			1,150(55.85%)	1,315(69.43%)	569(77.00%)		
Education level									
Kindergarten	247(2.51%)	9,591(97.49%)	31838.58	<0.001	41(0.64%)	80(2.96%)	126(16.56%)	247.013	<0.001
Primary school	16,661(17.17%)	80,383(82.83%)			6,180(10.52%)	7,149(23.59%)	3,332(41.68%)		

Junior High school	19,070(47.53%)	21,049(52.47%)			6,804(36.60%)	8,797(53.98%)	3,469(66.32%)		
Senior High school	17,637(64.19%)	9,839(35.81%)			6,097(54.48%)	8,354(69.12%)	3,186(75.89%)		
Sex									
Male	25,483(28.00%)	65,513(72.00%)	663.18	<0.001	8,170(16.92%)	11,753(36.14%)	5,560(54.52%)	406.459	<0.001
Female	28,132(33.70%)	55,349(66.30%)			10,952(23.50%)	12,627(43.72%)	4,553(57.01%)		
Region									
Central Shaanxi	26,674(30.29%)	61,375(69.71%)	15.81	<0.001	9,349(19.32%)	12,144(40.10%)	5,181(55.24%)	344.551	<0.001
Northern Shaanxi	13,526(31.20%)	29,822(68.80%)			4,268(19.32%)	6351(39.22%)	2907(57.38%)		
Southern Shaanxi	13,415(31.14%)	29,665(68.86%)			5,505(22.55%)	5,885(39.43%)	2,025(54.14%)		
Ethic group									
Han	50,505(30.68%)	114,137(69.32%)	3.90	0.048	18,104(20.19%)	22,960(39.62%)	9,441(55.47%)	21.178	<0.001
Non-Han	3,110(31.62%)	6,725(68.38%)			1,018(19.51%)	1,420(41.11%)	672(57.73%)		
All	53,615(30.73%)	120,862(69.27%)			19,122(20.15%)	24,380(39.71%)	10,113(55.61%)		

STROBE Statement—Checklist of items that should be included in reports of ***cross-sectional studies***

	Item No	Recommendation	Page No
**Title and abstract**	1	(*a*) Indicate the study's design with a commonly used term in the title or the abstract	1
		(*b*) Provide in the abstract an informative and balanced summary of what was done and what was found	1
**Introduction**			
Background/rationale	2	Explain the scientific background and rationale for the investigation being reported	2
Objectives	3	State specific objectives, including any prespecified hypotheses	3
**Methods**			
Study design	4	Present key elements of study design early in the paper	3-4
Setting	5	Describe the setting, locations, and relevant dates, including periods of recruitment, exposure, follow-up, and data collection	3-4
Participants	6	(*a*) Give the eligibility criteria, and the sources and methods of selection of participants	3
Variables	7	Clearly define all outcomes, exposures, predictors, potential confounders, and effect modifiers. Give diagnostic criteria, if applicable	4-5
Data sources/ measurement	8*	For each variable of interest, give sources of data and details of methods of assessment (measurement). Describe comparability of assessment methods if there is more than one group	*4*
Bias	9	Describe any efforts to address potential sources of bias	3-4
Study size	10	Explain how the study size was arrived at	3
Quantitative variables	11	Explain how quantitative variables were handled in the analyses. If applicable, describe which groupings were chosen and why	4

Statistical methods	12	(*a*) Describe all statistical methods, including those used to control for confounding	5
		(*b*) Describe any methods used to examine subgroups and interactions	5
		(*c*) Explain how missing data were addressed	-
		(*d*) If applicable, describe analytical methods taking account of sampling strategy	-
		(*e*) Describe any sensitivity analyses	-
**Results**			
Participants	13*	(a) Report numbers of individuals at each stage of study—eg numbers potentially eligible, examined for eligibility, confirmed eligible, included in the study, completing follow-up, and analysed	6
		(b) Give reasons for non-participation at each stage	-
		(c) Consider use of a flow diagram	-
Descriptive data	14*	(a) Give characteristics of study participants (eg demographic, clinical, social) and information on exposures and potential confounders	4
		(b) Indicate number of participants with missing data for each variable of interest	4
Outcome data	15*	Report numbers of outcome events or summary measures	4
Main results	16	(*a*) Give unadjusted estimates and, if applicable, confounder-adjusted estimates and their precision (eg, 95% confidence interval). Make clear which confounders were adjusted for and why they were included	6-8
		(*b*) Report category boundaries when continuous variables were categorized	6-8
		(*c*) If relevant, consider translating estimates of relative risk into absolute risk for a meaningful time period	-
Other analyses	17	Report other analyses done—eg analyses of subgroups and interactions, and sensitivity analyses	6-8
**Discussion**			
Key results	18	Summarise key results with reference to study objectives	9
Limitations	19	Discuss limitations of the study, taking into account sources of potential bias or imprecision. Discuss both direction and magnitude of any potential bias	13
Interpretation	20	Give a cautious overall interpretation of results considering objectives, limitations, multiplicity of analyses, results from similar studies, and other relevant evidence	9-13
Generalisability	21	Discuss the generalisability (external validity) of the study results	13
**Other information**			
Funding	22	Give the source of funding and the role of the funders for the present study and, if applicable, for the original study on which the present article is based	14

*Give information separately for exposed and unexposed groups.

Note: An Explanation and Elaboration article discusses each checklist item and gives methodological background and published examples of transparent reporting. The STROBE checklist is best used in conjunction with this article (freely available on the Web sites of PLoS Medicine at http://www.plosmedicine.org/, Annals of Internal Medicine at

http://www.annals.org/, and Epidemiology at http://www.epidem.com/). Information on the STROBE Initiative is available at www.strobe-statement.org.
